# Accuracy and safety of navigated pedicle screw insertion in cervical spine fractures

**DOI:** 10.3389/fsurg.2026.1773142

**Published:** 2026-02-20

**Authors:** Jalal Mirzamohammadi, Tor Arnøy Austad, Vidar Stenset, Donata Iwona Biernat, Mads Aarhus, Eirik Helseth, Hege Linnerud

**Affiliations:** 1 Department of Neurosurgery, Oslo University Hospital, Oslo, Norway; 2 Department of Neuroradiology, Oslo University Hospital, Oslo, Norway; 3 Faculty of Medicine, University of Oslo, Oslo, Norway

**Keywords:** cervical, navigation, neurovascular injury, pedicle screw, spinal fracture, traumatic

## Abstract

**Background:**

Pedicle screw (PS) fixation provides superior biomechanical stability compared with lateral mass screw (LMS) fixation for unstable cervical spine fractures (CS-Fx) but is associated with a risk of neurovascular injury. Navigation systems have improved PS placement accuracy, although most published studies remain small and underpowered to assess rare complications.

**Objective:**

To evaluate the accuracy and safety of navigation-assisted PS fixation for unstable CS-Fx in a population-based cohort.

**Methods:**

All consecutive patients with unstable CS-Fx who underwent navigated PS fixation at Oslo University Hospital between 2015 and 2024 were included in this study. Navigation was performed using preoperative CT-based surface matching. Postoperative CT scans obtained within 24 h were used to grade PS accuracy as Grade 1 (<2 mm breach), Grade 2 (2–4 mm), or Grade 3 (>4 mm). Complications related to PS placement were recorded.

**Results:**

A total of 345 patients (median age 68 years; 75% males) underwent fixation with 1,347 navigated PSs. Screw accuracy was Grade 1 in 90% of cases, Grade 2 in 8% of cases, and Grade 3 in 2% of cases. Surgery-related complications occurred in 23 patients (6.7%), of whom 11 experienced complications directly related to PS placement. The per-screw complication risk was 0.8%, increasing with decreasing accuracy: 0.1% (Grade 1), 6% (Grade 2), and 14% (Grade 3). Vertebral artery injury occurred in seven patients; two patients experienced new-onset nerve root injury, one had a misplaced screw breaching the atlanto-occipital joint, and one developed significant perioperative bleeding. No cases of new-onset spinal cord injury or screw pull-out were observed. Surgical site infections occurred in 3.5% of patients and were successfully treated with debridement and antibiotics in all cases, without the need for implant removal.

**Conclusion:**

Navigated cervical PS fixation is accurate and associated with a low rate of serious complications. Meticulous planning and surgical technique remain essential despite the use of navigation assistance.

## 1 Introduction

Unstable cervical spine fractures (CS-Fx) frequently require instrumented fusion to restore biomechanical stability. Several fixation techniques are available. Compared with lateral mass screw (LMS) fixation, pedicle screw (PS) fixation offers superior biomechanical strength (1, 2). However, traditional PS placement has been associated with an increased risk of injury to neurovascular structures, limiting its widespread use.

The introduction of navigation technologies in spine surgery has facilitated more accurate and safer PS placement, leading to broader adoption of PS fixation in the management of cervical spine fractures (3–5). Recent studies have indicated that navigated PS insertion in CS-Fx is associated with high accuracy and a low incidence of severe complications (6, 7). Nevertheless, most published series include analyses of fewer than 200 patients, providing limited statistical power to assess rare but serious complications (8–11). Larger cohort studies are therefore needed to provide a more robust evidence base for future systematic reviews and meta-analyses.

In this study, we present a consecutive cohort of 345 patients with unstable CS-Fx treated surgically using navigation-guided fixation. A total of 1,347 navigated screws were inserted, including C1 lateral mass screws, C2 pars interarticularis screws, C2 and subaxial PSs, and upper thoracic PSs. The primary outcomes were screw placement accuracy and procedure-related complications associated with navigated PS fixation.

## 2 Materials and methods

Oslo University Hospital (OUH) serves as the sole neurotrauma center in Southeast Norway, where all trauma-related cervical procedures for this population are performed. The population of Southeast Norway increased from 2.9 million in 2015 to 3.1 million in 2024. Norway has a publicly financed healthcare system characterized by equal access to care.

This study is a retrospective cohort analysis based on prospectively collected data from the Oslo Cervical Spine Fracture Registry, a quality-control database maintained by the Department of Neurosurgery at OUH (DPO approval no. 2014/12304). Since 2015, all residents of the southeastern region of Norway with a Norwegian social security number and an imaging-verified CS-Fx have been prospectively registered (12).

In 2015, we implemented the Brainlab spine navigation system (Brainlab AG, Munich, Germany), integrating preoperative cervical CT scans for navigated PS insertion. The registry includes demographic data, information of fracture morphology and management strategy, surgical details, and information on the use of intraoperative navigation for PS placement, screw placement accuracy, and surgery-related complications.

Between 2015 and 2024, a total of 4,689 patients with CS-Fx were registered in the Oslo Cervical Spine Fracture Registry. Of these, 1,014 (22%) underwent open surgical treatment. Among the surgically treated patients, 973 (96%) underwent surgery at OUH, while 41 (4%) were injured outside our health region and underwent primary surgery elsewhere.

**Figure 1:**
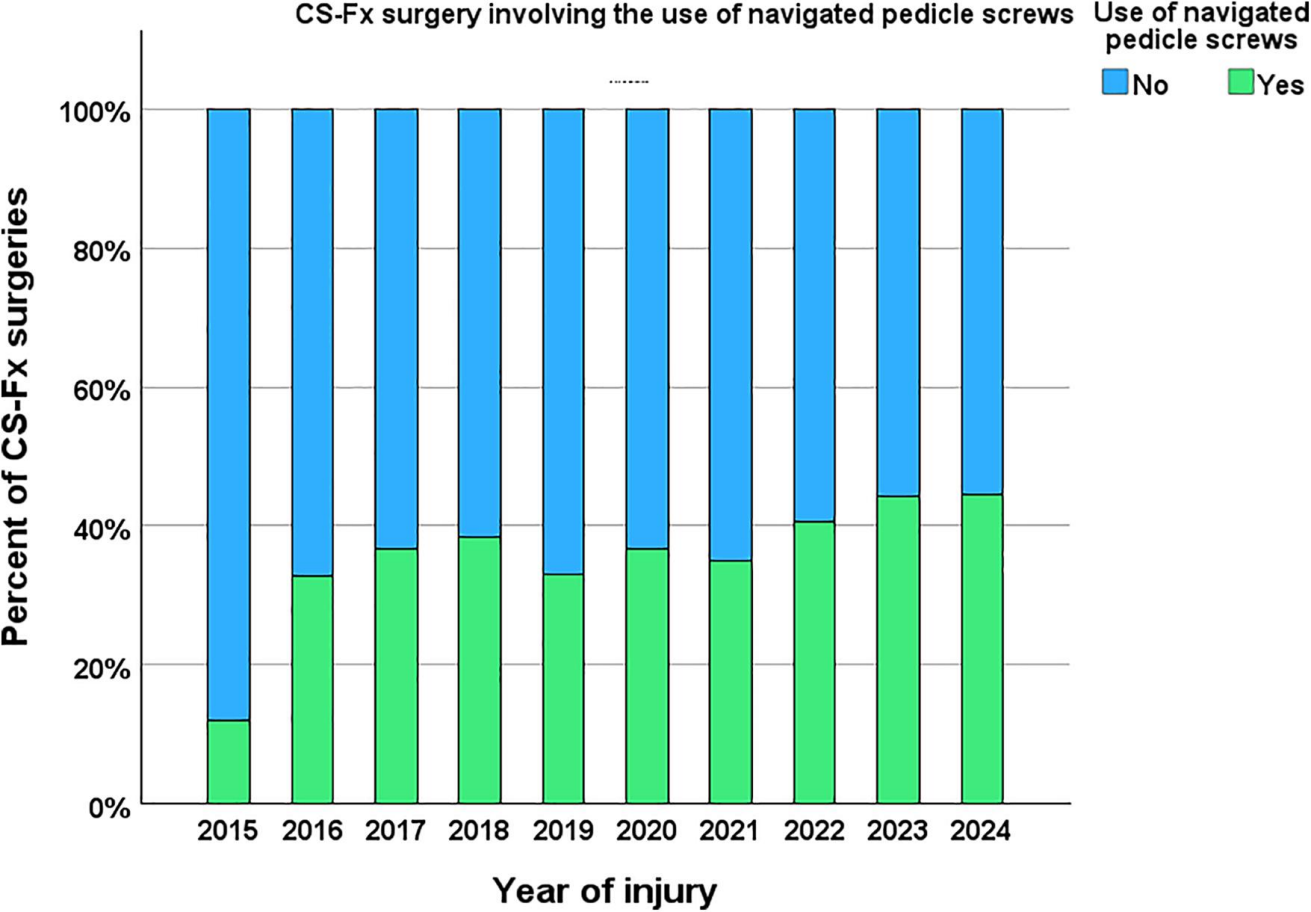
Number of surgical fixations for cervical spine injury performed at OUH during 2015–2024, without (blue) and with (green) navigated pedicle screws. Navigated PS was used in 347/973 (36%).

Routine preoperative imaging included cervical CT, cervical MRI, and CT angiography (CTA). Navigation-guided pedicle screw (PS) fixation was used in 347 of 973 (36%) surgically treated CS-Fx patients at OUH (Figure 1). Intraoperative three-dimensional imaging was not available; navigation was performed using surface matching based on preoperative CT scans.

During surgery, preoperative cervical CT images with a slice thickness of 0.625 mm were uploaded into the navigation system. The navigation reference array was rigidly attached to the spinous process at the level planned for pedicle screw (PS) insertion. Level-specific registration was performed using surface matching with multiple points, ensuring adequate spatial distribution and height variation. Registration accuracy was verified by correlating navigated pointer positions with known anatomical landmarks. A navigated drill was then used to create the pedicle entry tract prior to screw insertion. Screw length was determined intraoperatively by advancing the navigation ruler to the pointer tip. This workflow was repeated for each instrumented level. In cases with two adjacent, fully ankylosed, uninjured levels, a single registration was used to place navigated screws at both levels.

Postoperative cervical CT scans were obtained within 24 h for 345 of 347 patients. Two patients died within 24 h of surgery and therefore did not undergo postoperative CT; both were excluded from the cohort, and their deaths were deemed unrelated to the use of navigated PS.

The final study cohort consisted of 345 patients with unstable CS-Fx treated with navigated PS fixation at OUH between 2015 and 2024, all of whom had postoperative CT images available for assessment of screw placement accuracy.

**Figure 2:**
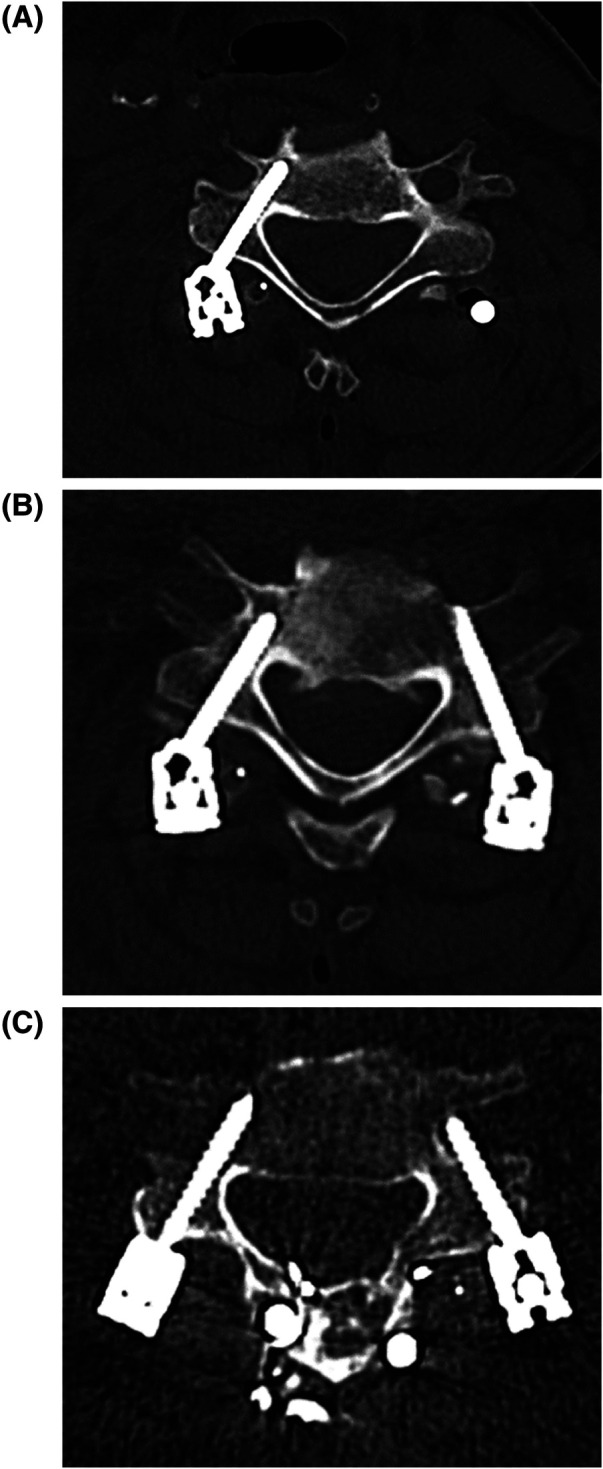
Accuracy of navigated PS placement on postoperative cervical CT. **(A)** Right side: PS level C5; accuracy Grade 1. **(B)** Left side: PS level C6; accuracy Grade 2; right side: PS level C6; accuracy Grade 1. **(C)** Left side; PS level C5; accuracy grade 3; right side: PS level C5; accuracy Grade 2.

Data were extracted from the registry and supplemented with medical charts and imaging reviews when necessary. The accuracy of PS placement in the thoracolumbar spine is typically assessed using the Gertzbein–Robbins scale (13). However, this scale is less suitable for evaluating screw placement in the smaller pedicles of the cervical spine. Therefore, in this study, PS placement accuracy was graded on postoperative CT scans as follows: Grade 1, pedicle perforation <2 mm; Grade 2, pedicle perforation 2–4 mm; and Grade 3, pedicle perforation >4 mm (Figure 2). This grading system aligns with that used in a recent systematic review and meta-analysis, which included 4,697 navigated cervical PSs from 18 studies (14). The CT-based Grade 1–3 classification was independently performed by five neurosurgeons (JM, TA, VS, EH, and HL) under the supervision of a neuroradiologist (DB). The 345 patients were allocated among the five neurosurgeons for evaluation. In instances of diagnostic uncertainty, the neuroradiologist and the senior spine neurosurgeon (HL) were consulted to reach a consensus. The assessments were not blinded, and inter- or intra-observer reliability analyses were not undertaken.

The primary outcome measures were as follows:

Accuracy of navigated PS placement, assessed on postoperative cervical CT obtained within 24 h after surgery, and
Complications associated with navigated PS fixation, including cervical spinal cord injury (cSCI), vertebral artery (VA) injury, nerve root injury, cerebrospinal fluid (CSF) leakage, surgical site infection, and revision surgery due to misplaced or dislodged PS.

If a VA injury was suspected intraoperatively or if postoperative CT imaging suggested that a screw trajectory might conflict with the VA course, CTA was performed immediately. Patients with CTA–verified VA injury received subcutaneous low-molecular-weight heparin (LMWH) during the acute phase, followed by oral acetylsalicylic acid therapy for a minimum of three months. A follow-up CTA was performed at one week and three months. In cases of clinical suspicion of cerebral infarction during the acute phase or follow-up period, a cerebral CT or MRI was obtained. Patients with nerve root injury were assessed for rehabilitation needs and received outpatient follow-up for minimum three months or as long as clinically indicated.

### 2.1 Statistical analysis

Descriptive statistics were applied to summarize the data. Categorical variables are presented as counts and percentages. Continuous variables are reported as the mean ± standard deviation (SD) for normally distributed data or median with interquartile range (IQR) for skewed data. Comparisons of complication risk across the three screw-accuracy groups were performed using Fisher's exact test. A *p* value < 0.05 was considered to indicate statistical significance. Confidence intervals not provided.

#### 2.1.1 Ethics

The study was approved by the Data Protection Officer (DPO) at OUH (approval no. 20/22933) and by the Regional Committee for Medical and Health Research Ethics (REC; approval no. 173594). Data were obtained from a hospital-approved quality registry for acute cervical spine fractures (OUH DPO approval no. 2014/12304). The requirement for individual patient consent was waived by both the DPO and REC.

## 3 Results

The study cohort comprised 345 patients who underwent posterior fixation for CS-Fx at OUH using navigated PSs, all of whom had postoperative cervical CT images available for accuracy assessment. The median patient age was 68 years (range 5–92 years; IQR, 57–75); 60% were ≥65 years, 75% were male, 48% had severe systemic disease (ASA score ≥3), and 73% had a fall-related injury (Table 1).

**Table 1:** Characteristics of patients with CS-Fx undergoing open surgical fixation with navigated pedicle screws (*N* = 345).

Characteristics	*N* (%)
Age (years)	
0–19	14 (4.1)
20–34	19 (5.5)
35–49	28 (8.1)
50–64	78 (22.6)
65–79	159 (46.1)
≥ 80	47 (13.6)
Sex	
Male	258 (74.8)
Preinjury ASA score	
≥ 3 Severe systemic disease	164 (47.5)
Preinjury living	
Dependent	36 (10.4)
Injury mechanism	
Fall	250 (72.5)
Motor vehicle	32 (9.3)
Bicycle	17 (4.8)
Sport	14 (4)
Other	22 (6.4)
Multiple trauma	
Yes	146 (42.3)
Main level of traumatic instability	
C0–C2	71 (20.6)
C2/C3	11 (3.2)
C3/C4	12 (3.5)
C4/C5	28 (8.1)
C5/C6	87 (25.2)
C6/C7	107 (31)
C7/Th1	29 (8.4)
Spinal cord injury - preoperative	
Yes	120 (34.8)
Cervical radiculopathy - preoperative	
Yes	61 (17.7)
VA injury – preoperative	
Yes	53 (15.4)

CS-Fx, cervical spine fracture; ASA, American Society of Anesthesiologists; VA, vertebral artery.

In descending order, the most frequent levels of instability were C6/C7 (31%), C5/C6 (25%), and C0–C2 (21%) (Table 1). At admission, 35% of patients had trauma-associated cSCI, 18% had cervical radiculopathy, and 15% had VA injury.

All 345 patients underwent posterior fixation using one or more navigated PSs. Posterior decompression (laminectomy) was performed in 102 patients (30%), and additional anterior fixation was performed in 98 patients (28%).

A total of 1,347 navigated pedicle PSs were inserted across 345 patients, ranging from 1 to 14 screws per patient, with a mean of 3.9 navigated PSs per patient. In most cases, the osteosynthesis construct involved a combination of navigated PS with non-navigated LMS. Navigated PSs were most frequently placed at C7, followed by T1, C2, C1, C6, and T2 (in descending order) (Table 2). At the C3–C5 levels, navigated PSs were used less frequently, as freehand LMSs were preferred due to the small pedicle diameter. The most caudal injured level in this cohort was C7/T1 (Table 1); thus, stabilization rarely extended to T3 or T4, which explains the low number of navigated PSs placed at these levels.

**Table 2:** Vertebral distribution and precision according to the modified Gertzbein–Robbins scale of navigated screws (*N* = 1,347 PS).

Variables	PS, *n* (%)	Precision navigated PS			Injury, *n*			Redo surgery, *n*
		Grade 1 *n* (%)	Grade 2 *n* (%)	Grade 3 *n* (%)	VA	SCI	Root	
C1	129 (100)	122 (94.6)	2 (1.5)	5 (3.9)	1	0	1	1
C2	193 (100)	181 (93.8)	10 (5.2)	2 (1.0)	2	0	0	0
C3	6 (100)	4 (66.7)	0 (0.0)	2 (33.3)	0	0	0	0
C4	6 (100)	5 (83.3)	0 (0.0)	1 (16.7)	1	0	0	0
C5	42 (100)	27 (64.3)	12 (28.6)	3 (7.1)	1	0	0	1
C6	123 (100)	95 (77.2)	25 (20.3)	3 (2.5)	2	0	0	1
C7	403 (100)	356 (88.4)	38 (9.4)	9 (2.2)	0	0	1	0
Th1	316 (100)	297 (94.0)	16 (5.1)	3 (0.9)	0	0	0	0
Th2	112 (100)	106 (94.6)	5 (4.5)	1 (0.9)	0	0	0	0
Th3	11 (100)	11 (100)	0 (0.0)	0 (0.0)	0	0	0	0
Th4	6 (100)	6 (100)	0 (0.0)	0 (0.0)	0	0	0	0
Sum, *n* (%)	1,347 (100)	1,210 (90)	108 (8)	29 (2)	7 (0.5)	0 (0)	2 (0.1)	3 (0.2)

PS, pedicle screw; VA, vertebral artery; SCI, spinal cord injury.

The placement accuracy was Grade 1 for 1,210 of the 1,347 screws (90%), Grade 2 for 108 (8%), and Grade 3 for 29 (2%) (Table 2). Suboptimal accuracy (Grade 2 or 3) was observed in 137 of the 1,347 screws (10.1%). The rate of suboptimal pedicle screw (PS) placement was high in the C3–C6 vertebrae (17%–36%), intermediate in C7 (12%), and low in C1–C2 and Th1–Th4 (≤6%) (Table 2).

A total of 23 of the 345 patients (6.7%) experienced complications (Table 3). Eleven of these complications were directly related to PS placement. Among these, the corresponding screw accuracy was Grade 1 in one patient, Grade 2 in six, and Grade 3 in four (Table 3). The estimated overall risk of a PS-related complication per screw was 0.8% (11/1,347). When stratified by accuracy, the risk of complications was 0.1% (1/1,210) for Grade 1 screws, 6% (6/108) for Grade 2 screws, and 14% (4/29) for Grade 3 screws (Fisher's exact test, *p* < 0.001).

**Table 3:** Complications associated with placement of the navigated PS.

Patient	PS	Accuracy	Injury	Consequence
1	C2	Grade 1	Perioperative bleeding	Circulatory collapse during surgery. Blood transfusion needed. Prolonged hospital stay.
2	C5dx and C6dx	Grade 3	VA	The previously implanted PS was removed and replaced with LMS. Imaging revealed occlusion of the right VA, most likely secondary to arterial dissection. No symptoms and signs of VA-associated cerebral infarction in the acute phase or during follow up.
3	C2	Grade 2	VA	Imaging revealed stenosis of the right VA, most likely secondary to arterial dissection. No symptoms and signs of cerebral infarction in the acute phase or during follow up.
4	C1	Grade 2	VA	Imaging revealed occlusion of the left VA, most likely secondary to arterial dissection. No symptoms and signs of cerebral infarction in the acute phase or during follow up.
5	C6	Grade 2	VA	Imaging revealed occlusion of the right VA, most likely secondary to arterial dissection. No symptoms and signs of cerebral infarction in the acute phase or during follow up.
6	C2	Grade 2	VA	Imaging revealed mild stenosis of the right VA, most likely secondary to compression from the screw and not arterial dissection. No symptoms and signs of cerebral infarction in the acute phase or during follow up.
7	C6	Grade 2	VA	Imaging revealed stenosis of the right VA, most likely secondary to arterial dissection. No symptoms and signs of cerebral infarction in the acute phase or during follow up.
8	C4	Grade 3	VA	Imaging revealed occlusion of the left VA, most likely secondary to arterial dissection. No symptoms and signs of cerebral infarction in the acute phase or during follow up.
9	C1	Grade 3	Nerve root	Pain and reduced sensation in dermatome corresponding to left C2-root. No need for specialized rehabilitation. Minor late effects.
10	C7	Grade 2	Nerve root	Pain and reduced sensation in dermatome corresponding to left C8-root. No need for specialized rehabilitation. Minor late effects.
11	C1dx	Grade 3	PS entering AO joint	Replaced PS in C1dx. No further surgery related complications.
12	C7 ×2 Th1 ×2	Grade 1 ×3 Grade 3 ×1	Infection	Antibiotics. Surgical revision. Screws and rods not removed. No late effects.
13	C1 ×2 C2 ×2	Grade 1 ×4	Infection	Antibiotics. Surgical revision. Screws and rods not removed. No late effects.
14	C5 ×2 C6 ×2 C7 ×2 Th1 ×2	Grade 1 ×6 Grade 2 ×2	Infection	Antibiotics. Surgical revision. Screws and rods not removed. No late effects.
15	C1 ×2 C2 ×2	Grade 1 ×3 Grade 2 ×1	Infection	Antibiotics. Surgical revision. Screws and rods not removed. No late effects.
16	C5 ×1 C6 ×2 C7 ×2	Grade 1 ×3 Grade 2 ×2	Infection	Antibiotics. Surgical revision. Screws and rods not removed. No late effects.
17	C1 ×2 C2 ×2	Grade 1 ×4	Infection	Antibiotics. Surgical revision. Screws and rods not removed. No late effects.
18	C7 ×2	Grade 1 ×2	Infection	Antibiotics. Surgical revision. Screws and rods not removed. No late effects.
19	C7 ×2 Th1 ×2	Grade 1 ×4	Infection	Antibiotics. Surgical revision. Screws and rods not removed. No late effects.
20	C1 ×2 C2 ×2	Grade 1 ×3 Grade 2 ×1	Infection	Antibiotics. Surgical revision. Screws and rods not removed. No late effects.
21	C7 ×2 Th1 ×2	Grade 1 ×4	Infection	Antibiotics. Surgical revision. Screws and rods not removed. No late effects.
22	C7 ×2 Th1 ×2	Grade 1 ×4	Infection	Antibiotics. Surgical revision. Screws and rods not removed. No late effects.
23	C1 ×2 C2 ×2	Grade 1 ×4	Infection	Antibiotics. Surgical revision. Screws and rods not removed. No late effects.

VA, vertebral artery; PS, pedicle screw; LMS, lateral mass screw; LMWH, low-molecular-weight heparin; AO, atlanto-occipital.

### 3.1 Cervical spinal cord injury

No patient experienced new-onset cSCI attributable to misplaced navigated PS.

### 3.2 Cerebrospinal fluid leakage

No patient experienced CSF leakage attributable to misplaced navigated PS.

### 3.3 Perioperative bleeding

One elderly patient with a type II odontoid fracture developed severe perioperative bleeding with circulatory collapse during posterior C1–C2 fixation (Harms technique), requiring blood transfusions and prolonged hospitalization. The indication for surgery was failure of a previously placed anterior odontoid screw. During dissection to prepare entry points for the C1 and C2 screws, profuse venous bleeding was encountered. Navigated screws were placed in C2, whereas freehand screws were inserted in C1 due to the intraoperative situation. This case is reported to ensure complete transparency regarding serious surgical complications, although the event was related to surgical exposure rather than the use of navigation.

### 3.4 Vertebral artery injury

VA injury associated with PS insertion occurred in seven patients and involved two Grade 3 and five Grade 2 screws (Table 3). This corresponds to an estimated risk of 2.0% per patient (7/345) and 0.5% per screw (7/1,347). All VA injuries were verified by CTA. None of the affected patients exhibited clinical symptoms or imaging evidence of cerebral or cerebellar infarction. One patient underwent revision surgery in which the misplaced navigated screws at C5 and C6 (right side) were removed and replaced with lateral mass screws.

### 3.5 Nerve root injury

Two patients (one with a Grade 2 and one with a Grade 3 PS) developed new-onset postoperative radicular pain and numbness without motor deficit, corresponding to suboptimal PS placement. One patient had symptoms in the left C2 dermatome, and the other had symptoms in the left C8 dermatome (Table 3). Neither underwent revision surgery for screw repositioning, and both improved over time.

### 3.6 Redo surgery for suboptimal screw placement

Revision for suboptimal PS placement was performed for 0 of the 108 Grade 2 screws and 3 of the 29 Grade 3 screws. As previously mentioned, one patient with a PS-associated vertebral artery injury underwent revision surgery in which two Grade 3 screws at C5 and C6 (right side) were removed and replaced with lateral mass screws. Another patient who had undergone posterior fixation from C1–C4 for a C2 fracture required revision due to a Grade 3 C1 screw that breached the atlanto-occipital (AO) joint.

### 3.7 Surgical site infection

Revision surgery due to surgical site infection was required in 12 of 345 patients (3.5%). All patients underwent surgical debridement without implant removal and received intravenous antibiotics, with complete recovery and no permanent sequelae.

No cases of navigated PS pull-out were observed.

## 4 Discussion

In our cohort of 345 patients, among the 1,347 inserted navigated PSs, the placement accuracy was satisfactory (Grade 1) for 90%, and suboptimal for 10% (grade 2 for 8%, and grade 3 for 2%). Surgery-related complications occurred in 23/345 (7%) patients. Among the complications, 11/23 were direct PS-associated complications (7 cases of VA injury, 2 cases of nerve root injury, 1 case of perioperative hemorrhage, and 1 case of screw misplacement in the AO joint). The twelve postoperative infections were not directly linked to the PS insertion, but rather to the surgical procedure itself. There were no cases of new-onset SCI and no cases of PS pull-out.

The superiority of PSs compared with LMSs in terms of pullout strength and biomechanical stability has been well-documented in both cadaver and clinical studies (1, 2, 7). Although PS placement has traditionally been associated with technical difficulty and a risk of neurovascular complications, its use has become more common with the introduction of navigation tools (4, 5, 7). Most studies published thus far on neurovascular complications after navigated PS insertion are small and likely underpowered to provide reliable estimates of these rare events. Our single-center cohort is larger than most comparable published series (6–11).

For grading PS placement accuracy, we chose not to use the classical Gertzbein–Robbins scale in our study, as we considered it not ideal for assessing placement in the small pedicles of the cervical spine (13). Instead, we graded PS accuracy as follows: Grade 1, pedicle perforation <2 mm; Grade 2, pedicle perforation 2–4 mm; and Grade 3, pedicle perforation >4 mm. This grading system aligns with a recent systematic review and meta-analysis of 4,697 cervical PSs from 18 studies (14). Thus, our results can be included in future systematic reviews and meta-analyses of navigated PS for unstable CS-Fx, both because of the cohort size and the matching methodology of screw precision grading.

The accuracy of PS placement in our cohort was Grade 1 (satisfactory) for 90% of PSs, Grade 2 (suboptimal) for 8%, and grade 3 (misplaced) for 2%, which is somewhat inferior to that reported in a recent meta-analysis (14). Even studies on free-hand insertion techniques have reported better accuracy for perfectly positioned screws (14). However, focusing only on the accuracy of navigated PS placement may be overly simplistic. The main aim should be to minimize PS-related complications while achieving biomechanically sufficient osteosynthesis. In general, the placement of a PS when feasible results in a stronger biomechanical construct than LMS-only fixation and, in some cases, allows for a shorter construct.

In our patient cohort, surgery-related complications occurred in 7% of patients, but only half of these were directly related to navigated PS use. Complications directly related to navigated PS use were seen only in patients with Grade 2 and Grade 3 screws, except in one patient, in whom we observed profuse bleeding during dissection of the entry point. The estimated overall risk of a PS-associated complication per PS was 0.8%. The risk according to PS placement accuracy was 0.1% for Grade 1 screws, 6% for Grade 2 screws, and 14% for Grade 3 screws. Therefore, accuracy undoubtedly matters.

No patients in our cohort had new-onset SCI, but there were two cases of new-onset radiculopathy due to PS insertion. These patients did not undergo reoperation, and both recovered with no/minor neurological deficits.

The incidence of VA injury and the modalities for detecting PS-associated VA injury vary across studies. A systematic review by Soliman et al. (7) revealed an overall incidence of VA injury of 0.4% for PS and 0% for LMS when various techniques were used. Another systematic review by Yoshihara et al. (15) also demonstrated an increased risk of VA injury using PS compared to LMS, but the difference was small.

In this series, 2% of patients had VA injury secondary to PS insertion, and the risk of VA injury per navigated PS was 0.5%. All VA injuries in our cohort were severe stenosis/occlusions secondary to arterial dissections treated with subcutaneous LMWH in the acute phase, followed by peroral acetylsalicylic acid for at least 3 months. None of the patients had cerebral infarction in the acute phase or during a minimum follow-up period of 3 months. The possibility of massive hemorrhage, various degrees of vessel wall injury, such as dissection and pseudoaneurysm formation, and late-onset complications have been described in the literature (16). The reported risk of cerebral infarction after traumatic or iatrogenic VA injury is low (11, 17–20).

Several authors have described an increased risk of VA injury in the midsection of the cervical spine (C3–C5), characterized by thin pedicles and a medially oriented screw trajectory (21). Tomasino et al. (22) studied individual variations in cervical bony anatomy and the VA course. The mean pedicle diameter increased from 4.9 mm to 6.5 mm from C3 to C7, and the pedicles were larger in male patients. The VA entered the transverse foramen at C6 in 82%, C5 in 13%, C7 in 3%, and C4 and C3 in 1%. Unusual pedicle anatomy or an irregular VA pathway was seen in 24% of patients. This emphasizes the need for a careful evaluation of both CT and CTA before surgery.

Although the risk of neurovascular injury from PS placement is low, this risk must, in our opinion, be considered and balanced individually for each level/screw during preoperative planning. Importantly, the cervical musculature in a tight surgical field tends to force the screwdriver medially and the screw tip laterally, a purely technical factor not eliminated by the use of navigation (23, 24).

Deep surgical site infection is a feared complication with potentially severe consequences for patients and increased costs for society. Known risk factors for surgical site infections in spine surgery include the duration of surgery, instrumentation, CSF leakage, obesity, and chronic steroid use (25). Our infection rate was 3.5%, which is well in line with other publications reporting infection rates ranging from 1.3%–8.8% (26–30). All 12 patients recovered after surgical debridement and antibiotic treatment without implant removal. In the absence of radiological signs of screw loosening, treatment of surgical site infection with irrigation, debridement and antibiotics without implant removal is reported to be a valid and effective option (31).

The suboptimal accuracy of our navigated PS placement for 10% of screws in our study warrants some reflection. The accuracy of spinal navigation based on preoperative CT scans is dependent on reliable surface matching of the posterior aspect of the vertebra. The quality of surface matching depends on the spread of registration points, the surface area of the exposed lamina/spinous process, and the contours of the lamina surface. Thus, a higher risk of error in vertebras C1, C3, C4, C5, and C6 is inherent. Another limitation of this navigation method is that the posterior part of the registered/navigated vertebral level must be intact and attached to the anterior column. If there is any undetected injury separating the anterior and posterior anatomy, this will constitute a major possible source of error. We always register each vertebra, except in total ankylosed spines with two neighboring uninjured vertebral levels, to avoid the “excursion phenomenon” (inaccuracy caused by differences in spinal alignment in the perioperative prone vs. supine position during preoperative CT scans). Finally, the reference clamp can be dislocated during surgery. Preoperative planning for navigated PS should address fracture morphology, bone density, spine degeneration, pedicle size and shape, and the course of the VAs.

There are numerous technical aids for the placement of PSs, ranging from basic free-hand techniques with 2D fluoroscopy to navigation by surface matching based on preoperative CT, intraoperative CT or 3D fluoroscopy, patient-specific 3D-printed templates, and robot-assisted surgery. Freehand 2D fluoroscopy-guided techniques for PS placement in the cervical spine require experience and can be especially demanding when the anatomy is altered by trauma or degeneration, in pediatric patients and in patients with anomalies. 2D fluoroscopy has significant limitations in the caudal cervical segments and cervicothoracic junction (6, 22). Intraoperative CT or 3D fluoroscopy provides a better view of this region. Recent studies comparing freehand and various navigation-assisted techniques have reported that the use of navigation-assisted techniques both increases accuracy and decreases radiation exposure to patients and staff (6, 32).

### 4.1 Strengths and limitations

This is a contemporary, population-based study conducted within a publicly funded healthcare system that ensures equal access to care. All patients underwent postoperative cervical CT to assess the accuracy of PS placement, accompanied by CTA when indicated by radiological or clinical findings.

Neurovascular complications following navigated PS insertion are rare; therefore, even a cohort of this size is insufficient on its own to provide precise risk estimates. Nevertheless, our series represents one of the largest to date and may contribute valuable data to future systematic reviews and meta-analyses.

The risk of suboptimal accuracy of navigated PS was highest in the C3 – C6 vertebrae. Most likely, this has resulted in a selection bias for LMS at these levels.

This study spans the period 2015–2024, during which the Brainlab spine navigation system was implemented in 2015. Consequently, nearly all PS inserted throughout the study period were placed using navigation. As a result, a meaningful comparison between posterior constructs with navigated PS and those without navigation within this time frame is not feasible.

This study was not designed to allow a meaningful comparison between posterior constructs using navigated pedicle screws and those relying exclusively on lateral mass screws, as navigated pedicle screws were preferentially used whenever anatomically and clinically feasible.

## 5 Conclusion

In this population-based cohort, navigated cervical PS fixation demonstrated high accuracy and a low rate of serious neurovascular complications. Although the placement of 10% of the screws was graded as suboptimal, the overall complication rate was low, and no patients developed new-onset spinal cord injury or screw pull-out. The risk of PS-related complications increased markedly with decreasing screw accuracy, underscoring the critical importance of meticulous preoperative planning and intraoperative technique. Navigation substantially facilitates safe PS placement but cannot eliminate anatomical and technical challenges inherent to the cervical spine. Our results add robust data to the literature and will be valuable for future systematic reviews and meta-analyses evaluating the safety and accuracy of navigated PS fixation for unstable cervical spine fractures.

## Data Availability

The raw data supporting the conclusions of this article will be made available by the authors, without undue reservation.
